# Differences in Expression Level of Helios and Neuropilin-1 Do Not Distinguish Thymus-Derived from Extrathymically-Induced CD4^+^Foxp3^+^ Regulatory T Cells

**DOI:** 10.1371/journal.pone.0141161

**Published:** 2015-10-23

**Authors:** Edyta Szurek, Anna Cebula, Lukasz Wojciech, Maciej Pietrzak, Grzegorz Rempala, Pawel Kisielow, Leszek Ignatowicz

**Affiliations:** 1 Center for Biotechnology and Genomic Medicine, Georgia Regents University, Augusta, Georgia, United States of America; 2 Mathematical Biosciences Institute, College of Public Health, Ohio State University, Columbus, Ohio, United States of America; 3 Ludwik Hirszfeld Institute of Immunology and Experimental Therapy, 53–114 Wroclaw, Poland; Institut Pasteur, FRANCE

## Abstract

Helios transcription factor and semaphorin receptor Nrp-1 were originally described as constitutively expressed at high levels on CD4^+^Foxp3^+^ T regulatory cells of intrathymic origin (tTregs). On the other hand, CD4^+^Foxp3^+^ Tregs generated in the periphery (pTregs) or induced *ex vivo* (iTregs) were reported to express low levels of Helios and Nrp-1. Soon afterwards the reliability of Nrp-1 and Helios as markers discriminating between tTregs and pTregs was questioned and until now no consensus has been reached. Here, we used several genetically modified mouse strains that favor pTregs or tTregs formation and analyzed the TCR repertoire of these cells. We found that Tregs with variable levels of Nrp-1 and Helios were abundant in mice with compromised ability to support natural differentiation of tTregs or pTregs. We also report that TCR repertoires of Treg clones expressing high or low levels of Nrp-1 or Helios are similar and more alike repertoire of CD4^+^Foxp3^+^ than repertoire of CD4^+^Foxp3^-^ thymocytes. These results show that high vs. low expression of Nrp-1 or Helios does not unequivocally identify Treg clones of thymic or peripheral origin.

## Introduction

Regulatory CD4^+^Foxp3^+^ T cells (Tregs) play an indispensable role in maintaining homeostasis of the immune system by preventing autoimmunity and by controlling the strength and duration of immune responses against a variety of self and non-self antigens [1, 2]. Tregs can be divided into two major populations according to their cellular origin: tTregs, which develop from CD4^+^CD8^+^ thymocytes in the thymus and pTregs, which arise by conversion from conventional CD4^+^Foxp3^-^ T cells in peripheral tissues [3]. Both subsets share similar molecular and phenotypic signatures, including high expression of Foxp3, CD25, CTLA-4, GITR, ICOS, CD103, low expression of CD127, a broad TCR repertoire, and use various suppressive mechanisms to control effector cells [3]. However, the basic questions concerning the proportions of tTregs and pTregs in different organs and whether these subsets represent “more of the same” or differ in function and/or antigen specificities have not been satisfactorily clarified thus far [4, 5]. This information is critically important for the design of clinical protocols that will either expand preexisting tTregs or accelerate *de novo* conversion to pTregs. Because mice with impaired tTregs development suffer from multiorgan autoimmunity [6–8], whereas aged, pTreg-deficient mice develop allergic inflammation in the small intestine and have increased rates of preeclampsia [9, 10], Tregs of different origin may play non-redundant roles in controlling autoimmunity [4]. It has also been proposed that tTregs control tolerance to self-antigens because their differentiation in the thymus is guided by TCRs that recognize self-antigens with relatively high affinities [11, 12]. On the other hand, pTregs may represent clones with TCRs specific for foreign antigens derived from commensal microbiota, diet and various pathogens [13–15]. Comprehensive analysis of tTregs specificities showed that tTregs and pTregs can recognize both self and non–self antigens [16–19]. Thus, to understand how pTregs recruitment complements tTregs induced peripheral tolerance to self and non-self antigens, it is desirable to have a reliable marker(s) discriminating Treg clones of different origin.

It was reported that tTregs, but not pTregs, constitutively express high level of Helios transcription factor [20]. Helios is a member of the Ikaros family of transcription factors, which regulate lymphocyte development, and almost all CD4^+^Foxp3^+^ thymocytes are Helios^high^ [3]. However, Helios deficiency does not affect development of tTregs or their survival, suggesting that Helios is not mandatory for tTregs lineage commitment [3]. It was also found that most CD4^+^CD8^+^ thymocytes that are Helios^high^ die upon negative selection [21]. This observation concurred with the current paradigm that thymic precursors of tTregs can withstand stronger TCR-mediated signals, but whether this feature is responsible for positive selection or reflects lower sensitivity to negative selection of tTregs remains controversial [22–24]. The physiological importance of Helios for tTregs function is also unclear because Helios-deficient Tregs had unimpaired immunoregulatory properties [3].

Neuropilin-1 (Nrp-1) is another molecule that was reported to be expressed at high levels on mouse tTregs but not on pTregs [25, 26]. Nrp-1 plays a diverse role during embryonic development in the vascular and neural systems and Nrp-1-deficient mice die prematurely [27]. However, mice with conditional Nrp-1 deficiency in T cells develop normally, and their thymic differentiation of tTregs proceeds unperturbed. Constitutively high expression of Nrp-1 on Tregs is not influenced by TCR activation but depends on TGFβ and is directly controlled by Foxp3 [28]. In the periphery, Nrp-1 expression boosts Tregs capacity to infiltrate tumors [29, 30], potentiates their suppressive activity by enhancing their clustering with dendritic cells (DCs), and participates in formation of immunological synapses [31]. Nrp-1 also improves Treg stability at inflammatory sites [32], supports conversion of naive CD4^+^ cells to pTregs and interferes with their differentiation to T helper 17 (Th17) cell lineage [33]. Thus, there is convincing experimental evidence that Nrp-1 expression on Tregs enhances these cells immunoregulatory properties, but whether its variable expression levels mark Tregs of different origins remains unclear.

There is a general consensus that most CD4^+^Foxp3^+^ thymocytes that are immediate precursors of tTregs have constitutively high expression of Helios and Nrp-1 [25]. However, different cues in the periphery can alter expression of these molecules on tTregs and pTregs. Akimova and colleagues, reported that expression of Helios is increased in all Tregs activated by self and non-self antigens outside the thymus [34], and Gottschalk and colleagues reported that *in vitro*-induced Tregs (iTregs) are Foxp3^+^Helios^high^, and that *in vivo* the type of APCs presenting antigen(s) to naive CD4^+^ T cells converting to pTregs influence the expression of Helios by pTregs [35]. It was also reported that the Foxp3^+^Helios^low^ subset comprises Tregs of different origin that encountered antigen sometime in the past but not recently, whereas Foxp3^+^Helios^high^ can be also generated *ex vivo* as iTregs [35]. In agreement with the last observation, Schliesser et al. showed that naive allogenic CD4^+^ T cells that converted to iTregs in the presence of TGFβ and retinoic acid or rapamycin were mostly Helios^high^, and half of these cells were also Nrp-1^high^ [36]. Furthermore, although high expression of Helios correlates well with high expression of Nrp-1 on tTregs, Helios^low^ Tregs contain a significant fraction of Nrp-1^high^ cells, particularly in the peripheral lymph nodes, probably because Nrp-1 can be induced by TGFβ and proinflammatory cytokines. Because Helios^−^Nrp-1^−^ iTregs can make IFN-γ and/or IL-17 in the proinflammatory milieu in the gut, it is possible that these cells are not stably committed Tregs, but instead can be reprogrammed into CD4^+^ effectors [37]. In view of above described observations it is clear that the elucidation whether variable levels of Helios and Nrp-1 on Tregs can be used as indicators of their intrathymic or extrathymic origin requires other, not yet exploited, experimental approaches.

Here, we analyzed expression levels of Nrp-1 and Helios by Tregs in various genetically modified mouse strains that have impaired development of tTregs or pTregs, and then compared the TCR repertoires of CD4^+^Foxp3^+^ Tregs expressing low vs. high levels of Nrp-1 or Helios to the TCR repertoires of CD4^+^Foxp3^+^ and CD4^+^Foxp3^-^ thymocytes, to estimate their relative similarity to each other. First, we show that absence of Aire-controlled self-peptides, which reportedly drive tTregs differentiation but that are unessential for pTregs generation, did not change the proportions of Tregs with low vs high expression of Nrp-1 or Helios. Second, we found no change in the expression profiles of Nrp-1 or Helios on intestinal Tregs thriving in the gut of mice unable to convert naïve, intestinal CD4^+^ cells to pTregs due to either abolished presentation of microbe and diet–derived antigens or a dysfunctional CNS1 region in the Foxp3 locus. Third, we found that the peripheral TCR repertoires of CD4^+^Foxp3^+^Nrp-1^high^ (or Helios^high^) and CD4^+^Foxp3^+^Nrp-1^low^ (or Helios^low^) cells were similar to each other and resembled the repertoire of CD4^+^Foxp3^+^ thymocytes, but both these repertoires were different from TCRs expressed by naive CD4^+^Foxp3^-^ cells. Altogether, these results indicate that high or low expression of Helios or Nrp-1 does not unambiguously discriminate between pTregs and tTregs.

## Results

### Expression of Nrp-1 and Helios by tTregs in mice lacking low-abundant self-peptides

**Fig 1:**
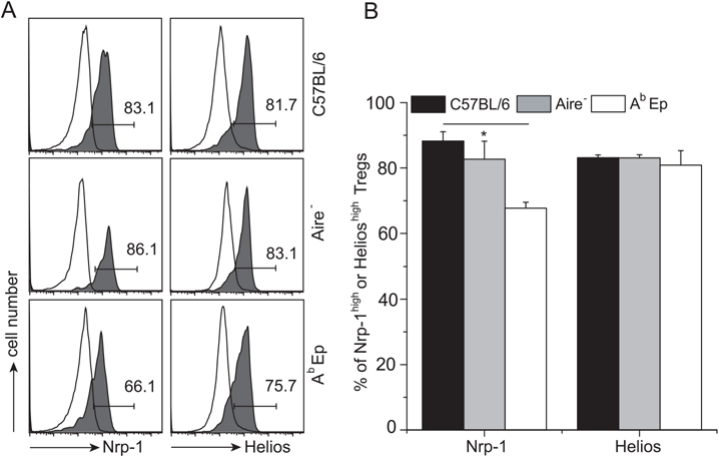
Expression of Nrp-1 and Helios on CD4^+^Foxp3^+^ thymocytes positively selected in the presence or absence of Aire controlled self-peptides. (A) Expression of Nrp-1 and Helios on CD4^+^Foxp3^+^ thymocytes from C57BL/6, Aire^-^ or “single peptide” A^b^Ep mice. The filled histograms show staining with specific antibodies, and the open histograms show staining with irrelevant IgG (also applicable to other figures). (B) Bars show the mean % of cells (+/- SD) expressing high level of indicated marker, and represent data from three to five mice. Gating for Nrp-1 and Helios stainings is shown in S2 Fig. For all data shown, numbers on plots show percentage of gated cells in the studied CD4^+^ subset, and statistical significance was calculated using Student’s *t* test (p<0.05). Total number of CD4^+^ and Tregs in analyzed strains is shown in S4A Fig.

Several lines of evidence suggest that tTregs differentiate from immature thymocytes which bind low-abundant, often tissue specific self-peptides with relatively high affinity [38, 39]. Therefore, we first compared the levels of Nrp-1 and Helios expression on CD4^+^Foxp3^+^ thymocytes in two strains of mice that lack intrathymic expression of many or all tissue-specific peptides. The first strain, Aire^-^, has impaired expression of these peptides because Aire controls their processing and expression by thymic medullary epithelial cells [40, 41]. The second strain, A^b^Ep, expresses class II MHC molecules (A^b^) bound exclusively with a single covalently linked peptide (Ep) [42]. The covalently linked Ep prevents presentation of endogenously processed self-peptides other than Ep, including Aire-controlled peptides, and eliminates the formation of peptide-specific thymic niches, which were suggested to facilitate differentiation of tTreg precursors [43]. As previously reported, (also see S1 Fig), both the aforementioned strains of mutant mice supported development of CD4^+^ thymocytes into CD4^+^Foxp3^-^ and CD4^+^Foxp3^+^ lineages [19, 44]. Fig 1A and 1B show that in Aire^-^ and in A^b^Ep mice the proportion of CD4^+^Foxp3^+^ thymocytes expressing high levels of Nrp-1 or Helios was similar to the proportions of these cells in wild type mice, although in A^b^Ep mice slightly lower proportion of CD4^+^Foxp3^+^Nrp-1^high^ could be observed. These results showed that elimination of self-peptides considered to play an essential role in positive selection of tTregs had no or little impact on the phenotype of CD4^+^Foxp3^+^ thymocytes with regard to the level of constitutive expression of Nrp-1 or Helios. Thus, it is unlikely that high expression of these two genes by developing CD4^+^Foxp3^+^ thymocytes depends on high affinity binding of the unique, positively selecting self-peptides.

### Nrp-1 and Helios expression on Tregs in mice unable to present non-self antigens from microbiota and diet in the colon

Colonic Tregs contain a higher proportion of Nrp-1^low^ and Helios^low^ clones [20, 25]. Because conversion of naive CD4^+^ T cells to pTregs occurs most frequently in mucosal organs, increased frequency of Nrp-1^low^ and Helios^low^ Tregs in the colon was explained by enhanced recruitment of pTregs upon contact with microbe or diet derived antigens [14, 45]. Therefore, we examined whether the abundance of colonic pTregs expressing low levels of Nrp-1 and/or Helios will change in “single peptide” A^b^Ep mice with impaired ability to present non-self antigens. Surprisingly, regardless of the inability to present commensals and food-derived antigens, there was no significant difference in expression profile of Nrp-1 on colonic Tregs in these mice (S3A and S3B Fig). This observation suggested that lack of microbe and diet antigens-specific recruitment of pTregs had little effect on the proportion of the Foxp3^+^Nrp-1^low^ colonic Tregs. In contrast, the proportion of Foxp3^+^Helios^high^ cells was increased in all peripheral organs (including colon) of A^b^Ep mice as compared to control mice (S3C and S3D Fig). This last observation suggested that abundant expression of the same auto-antigen in the thymus and in the periphery of “single peptide” A^b^Ep mice provides sustained, weak TCR-mediated signal to all Tregs, which supports constitutively high expression of Helios on these cells. This conclusion corresponds with the previous reports that Helios expression increases on recently activated Tregs and with an observation that the phenotype of genuine antigen-inexperienced CD4^+^Foxp3^-^ clones in A^b^Ep mice resembles the phenotype of chronically activated T cells [46, 47].

### Nrp-1 and Helios expression on Tregs in pTreg-deficient CNS1^mut^ mice

**Fig 2:**
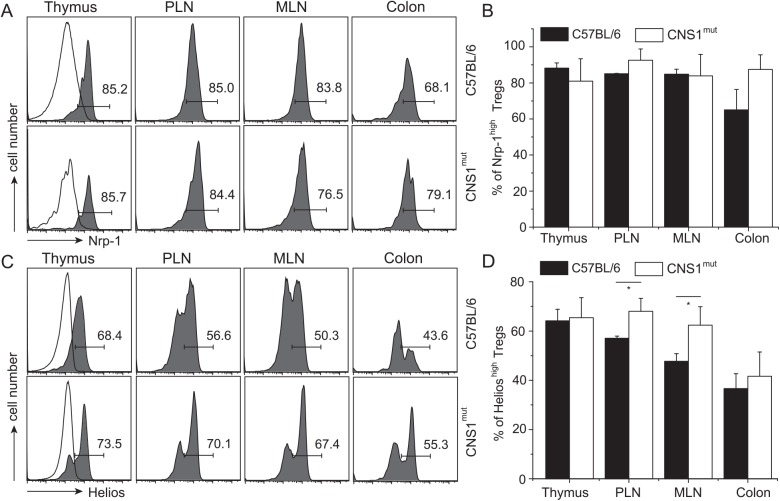
Expression of Nrp-1 and Helios on Tregs from CNS1^mut^ mice. Expression of Nrp-1 (A) and Helios (C) on CD4^+^Foxp3^+^ cells from C57BL/6 and CNS1^mut^ mice in indicated organs. (B, D) Bars show the mean % of cells expressing high level of indicated marker (+/- SD), and represent data from three to five mice. Total number of CD4^+^ and Tregs in analyzed strains is shown in S4B Fig.

Recently, it has been shown that conserved, non-coding sequence (CNS-1) in the Foxp3 promoter region containing TGFβ-NFAT response element is required for generation of pTregs [48]. Thus, CNS-1 deficient mice lack most of the TGFβ-induced pTregs, and with age become prone to allergic inflammation in the small intestine [9]. We therefore compared the proportions of CD4^+^Foxp3^+^ T cells expressing high and low levels of Nrp-1 and Helios from CNS1^mut^ deficient and C57BL/6 mice. Fig 2A shows that the proportions of Nrp-1^high^ to Nrp-1^low^ Tregs in all tested organs, including the colon, from control and CNS-1 mutant mice were not significantly different (Fig 2B). This indicated that CNS-1 ablation, which interferes with pTregs differentiation, had little or no influence on the proportions of CD4^+^Foxp3^+^Nrp-1^low^ vs CD4^+^Foxp3^+^Nrp-1^high^ cells. The proportions of Tregs expressing high and low levels of Helios in CNS1^mut^ mice as compared to normal mice were slightly changed in favor of the former population (Fig 2C), but these changes were not statistically significant (Fig 2D) in the colon. Overall, since CNS1-deficient mice are virtually devoid of pTregs, the above observations suggest that *in vivo* tTregs can express reduced levels of Nrp-1 and Helios.

### Impact of pro-inflammatory milieu on expression of Nrp-1 and Helios by Tregs

**Fig 3:**
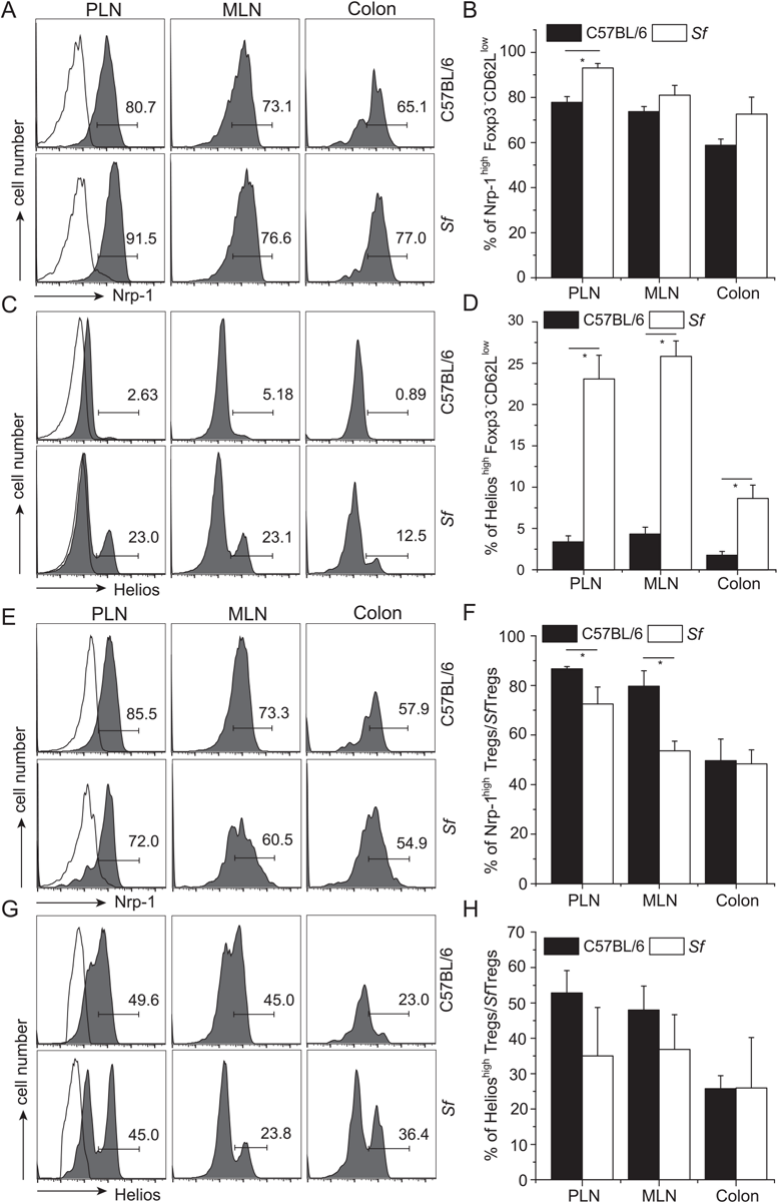
Expression of Nrp-1 and Helios on activated non-Treg and Treg cells in pro-inflammatory milieu. Expression of Nrp-1 or Helios on CD4^+^Foxp3^GFP-^CD62L^low^ cells (A-D) and on Tregs (GFP+) (E-H) from indicated organs of normal C57BL/6 and *scurfy* (*Sf*) mice. Bars (B, D, F, H) show the proportion (+/- SD) of cells and represent data from three to five mice. Total number of CD4^+^ and Tregs in analyzed strains is shown in S4C Fig.

It was proposed that in pro-inflammatory environment CD4^+^Foxp3^-^ T cells up-regulate Nrp-1 and Helios prior to their conversion to pTregs which maintain expression of these molecules at high levels [25, 34]. Therefore, to find whether higher proportion of Foxp3^-^ T cells expressing elevated levels of Nrp-1 and Helios translates into higher proportion of Tregs (i.e. Foxp3^+^) that constitutively express these markers, we examined the expression of Nrp-1 and Helios on CD4^+^Foxp3^-^CD62L^low^ T cells in *Sf*Foxp3^GFP^ *(scurfy)* mice, where most CD4^+^ T cells are activated by self-antigens, and in which Tregs lack functional Foxp3 but express the GFP reporter (*Sf*Tregs) [49, 50]. As shown in (Fig 3A–3D) *scurfy* mice had higher proportions of CD4^+^Foxp3^GFP-^CD62L^low^Helios^high^ and CD4^+^Foxp3^GFP-^CD62L^low^Nrp-1^high^ cells than normal mice, in all examined organs. In contrast, the proportions of Nrp-1^high^ or Helios^high^ *Sf*Tregs (GFP^+^) in *scurfy* mice were not higher or—as in case of Nrp-1^high^ cells in lymph nodes—significantly lower than in normal mice (Fig 3E–3H). These observations show that increased proportion of Tregs expressing high levels of Nrp-1 or Helios cannot be explained by conversion of higher number of Foxp3^-^ precursors expressing high levels of these molecules.

### Analysis of the TCR repertoires of Nrp-1^high^or Nrp-1^low^ and Helios^high^ or Helios^low^ Tregs

**Fig 4:**
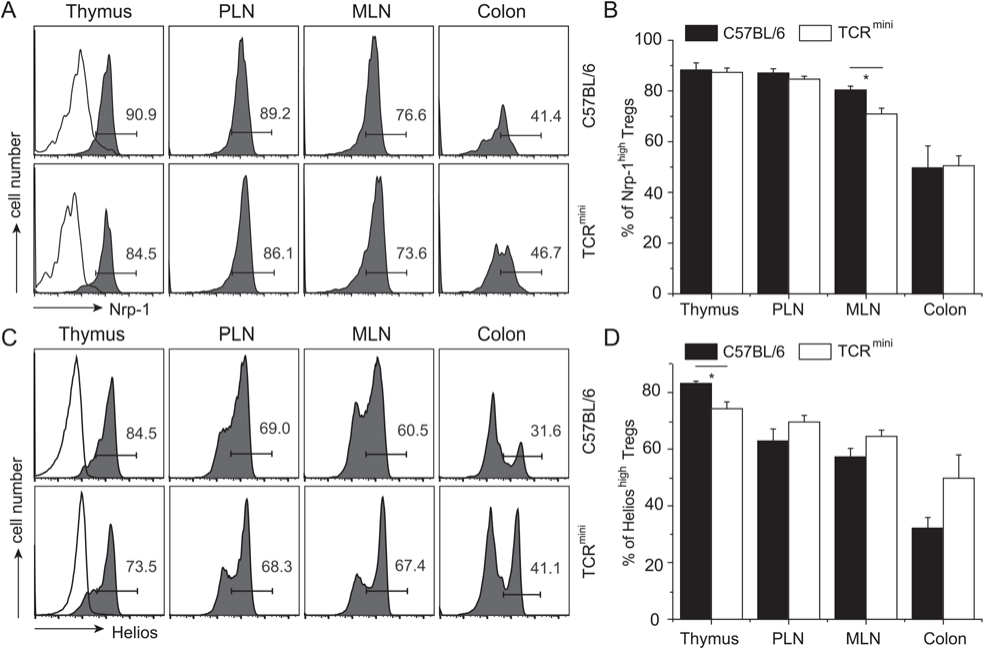
Proportions of CD4^+^Foxp3^+^ cells expressing high levels of Nrp-1 and Helios in TCR^mini^ vs C57BL/6 mice. Expression of Nrp-1 (A) and Helios (C) on CD4^+^Foxp3^+^ cells from C57BL/6 and TCR^mini^ mice in indicated organs. (B, D) Bars show the mean % of cells expressing high level of indicated marker (+/- SD), and represent data from three to five mice. Total number of CD4^+^ and Tregs in analyzed strains is shown in S4D Fig.

It has been proposed that progenitors of tTregs and pTregs, i.e. CD4^+^Foxp3^+^ vs CD4^+^Foxp3^-^ thymocytes, undergo positive selection within different windows of TCR sensitivity for the selecting self MHC/peptide complexes [22] and therefore, many TCRs frequently found on tTregs are rare or not found on naïve T cells and vice versa. This asymmetric allocation of TCRs is reflected in the peripheral repertoires of tTregs and pTregs, allowing one to deduce the origin of specific Tregs clones [51–53]. To compare the TCR repertoires of different CD4^+^ subsets we used TCR^mini^ mice in which a limited but diverse repertoire allows for a comprehensive analysis of TCRs on CD4^+^ T cells in various organs. The diversity of TCR repertoire in these mice can be studied by analysis of the diversity of the TCR-α chain only. The TCR^mini^ mice have a normal cellular composition of lymphoid organs [52], and Fig 4A and 4C show that proportions of Nrp-1^high^ or Helios^high^ cells among Tregs from various organs of TCR^mini^ and normal control mice were similar (Fig 4B and 4D). To determine how the TCR repertoires of Tregs with high or low expression of Nrp-1 or Helios correspond to TCR repertoires of CD4^+^Foxp3^+^ and CD4^+^Foxp3^-^ thymocytes, we compared the dominant TCRs expressed by Nrp-1^high^ vs Nrp-1^low^ and Helios^high^ vs Helios^low^ Tregs from colon or MLNs to CD4^+^Foxp3^+^ and CD4^+^Foxp3^-^ thymocytes. For this purpose, we sorted these cells according to expression of Foxp3 and high vs. low expression of Nrp-1 or Helios, amplified their TCR-α chains and sequenced their complementarity determining region 3 (CDR3) by high-throughput sequencing. We anticipated that if most tTregs exported to the periphery retain Helios^high^ and Nrp-1^high^ expression, whereas extrathymically generated pTregs remain Helios^low^ and Nrp-1^low^, the TCR repertoire of CD4^+^Foxp3^+^Nrp-1^high^ or CD4^+^Foxp3^+^Helios^high^ thymocytes will be similar to TCR repertoire of CD4^+^Foxp3^+^Nrp-1^high^ or CD4^+^Foxp3^+^Helios^high^ Tregs respectively, but will be mostly different from the TCR repertoires expressed by Nrp-1^low^ or Helios^low^ pTregs, which should resemble the repertoire of CD4^+^Foxp3^-^ thymocytes.

**Fig 5:**
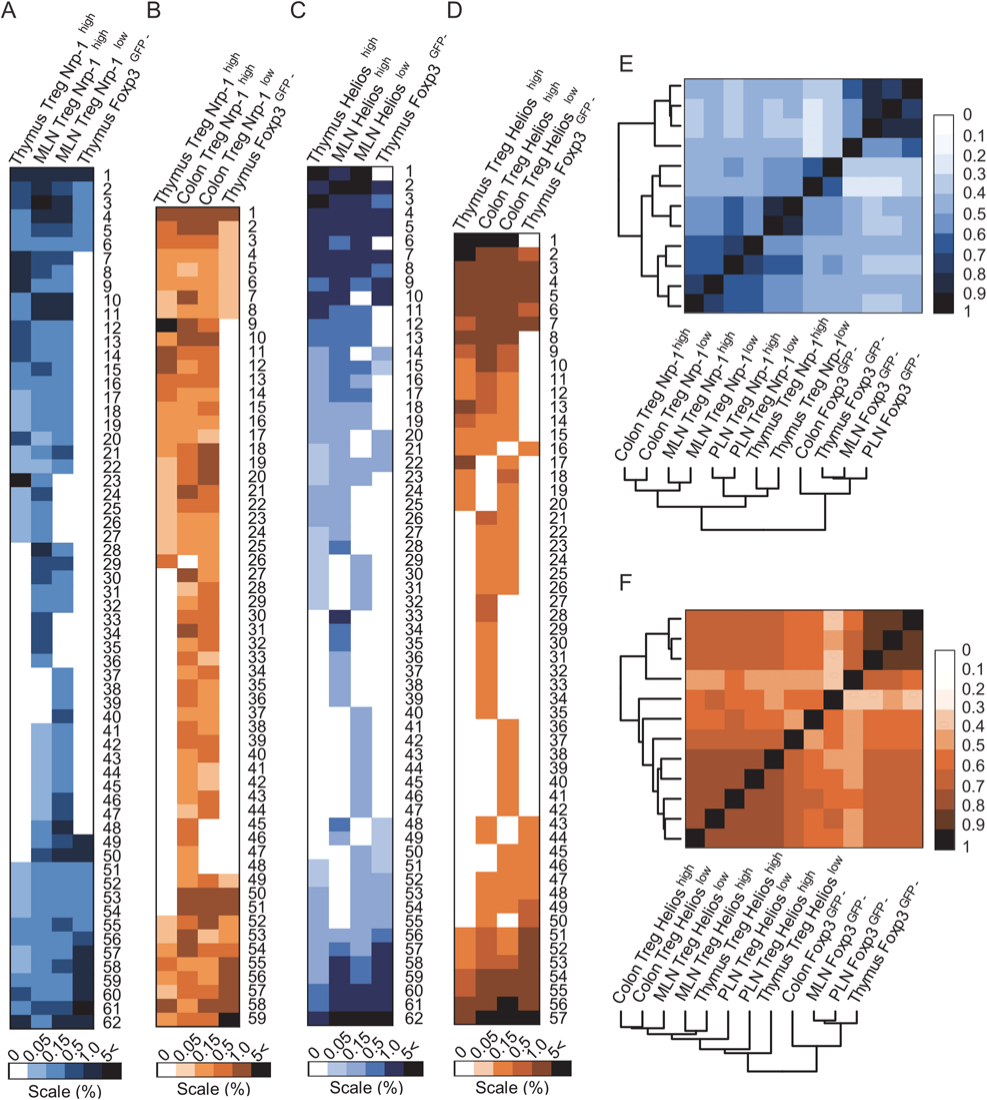
Distribution of dominant TCRs from intestinal CD4^+^Foxp3^+^ cells with high or low expression of Nrp-1 or Helios on different subsets of CD4^+^ thymocytes. Distribution of 50 most dominant TCRs from mesenteric (A, C) or colonic (B, D) CD4^+^Foxp3^+^ Nrp-1^high^ or Nrp-1^low^ (A, B) and CD4^+^Foxp3^+^ Helios^high^ or Helios^low^ cells (C, D) (two inner columns) on CD4^+^Foxp3^+^Nrp-1^high^ (A) or CD4^+^Foxp3^+^Helios^high^ (C) and on CD4^+^Foxp3^-^ thymocytes (A-D)(two outer columns). Color shades reflect the relative frequency of a given TCR in each organ. Sequences for heat maps (A-D) are listed in S5A–S5D Figs (respectively). (E, F) The hierarchical diagrams depict similarity indices (MII) for the whole TCR repertoires retrieved from Nrp-1^low^ or Nrp-1^high^ and from Helios^low^ or Helios^high^ subsets from indicated organs to the corresponding CD4^+^Foxp3^−^ subsets.

Fig 5 shows heat maps that depict the frequencies and distribution of 50 dominant TCRs from Nrp-1^high^ or Nrp-1^low^ (Fig 5A and 5B) and Helios^high^ or Helios^low^ (Fig 5C and 5D) Tregs from mesenteric lymph nodes (MLNs) or colon, and these TCR frequencies on the respective subsets of CD4^+^Foxp3^+^ or CD4^+^Foxp3^-^ thymocytes. Vast majority of TCRs retrieved from colonic Nrp-1^high^ or Nrp-1^low^ and Helios^high^ or Helios^low^ Tregs expressed the same TCRs and often at comparable frequencies. In fact, the TCR repertoires of Tregs with variable levels of Nrp-1 and Helios shared many dominant TCRs, of which a significant number was also found on CD4^+^Foxp3^+^ thymocytes. These results were also confirmed by statistical analysis where TCR repertoires expressed by Nrp-1^high^ or Nrp-1^low^ and Helios^low^ or Helios^high^ Tregs and CD4^+^Foxp3^+^ thymocytes clustered on separate branch of the diagram than naive, peripheral CD4^+^Foxp3^-^ populations retrieved from different lymphoid or mucosal organs (Fig 5E and 5F). Overall, these results indicate that the level of Nrp-1 or Helios expression does not mark different, peripheral Treg subsets and suggested that many of clones contained in these Treg subsets are shared, suggesting that these cells recognize the same spectrum of antigens.

## Discussion

In the present study, we used several approaches to examine whether different expression levels of Nrp-1 and/or Helios by Tregs marks their intrathymic and extrathymic origin. First, we analyzed the proportions of Tregs expressing high or low levels of these molecules in different genetically modified mouse strains, in which the differentiation of tTregs and pTregs was compromised due to (a) reduced diversity of self-peptides that are believed to drive selection of tTregs in the thymus (A^b^Ep and Aire^-^), (b) compromised presentation of microbial antigens in the mucosal tissues driving conversion to pTregs or (c) impaired regulation of Foxp3 which affects exclusively pTregs formation later in life (CNS1^mut^). In all of these mice, the proportions (and total number) of Tregs with low or high expression of Nrp-1 was approximately similar, suggesting that independently of the origin, Tregs do not remain imprinted with either low or high expression of Nrp-1. Our results also suggest that high expression of Nrp-1 is not a characteristic of as many tTregs as high expression of Helios, suggesting that of these two molecules, the high expression of the latter is more suitable to detect tTregs in certain circumstances. A similar conclusion has also been reached when expression of Nrp-1 and Helios by Tregs was analyzed in different inbred or outbred strains of mice or in humans [54] [55]. Likewise, in the Foxp3^IRES-RFP^ × BAC-Foxp3^Cre-GFP^ mice where pTregs and tTregs clones express distinctive pattern of fluorescent tags, many *ex vivo* or *in vivo* converted pTregs were consistently Nrp-1^high^, whereas most, though not all, *in vivo* converted pTregs were Helios^low^ [56]. However, Helios is expressed by fraction of *in vivo* antigen-primed CD4^+^Foxp3^-^ cells, and some of them may convert to pTregs in the presence of TGFβ [3].

To date, no unambiguous phenotypic marker distinguishing tTregs from pTregs or a definitive test that distinguishes their *in vivo* functions have been developed. So far, the most conclusive way to determine the origin of specific Treg clones is to sequence their TCRs and then extrapolate these to the repertoire of CD4^+^Foxp3^+^ thymocytes [16, 51–53, 57]. If these TCRs are found on the tTregs precursors, it strongly supports the scenario in which commitment of these cells to Treg lineage occurred during their intrathymic differentiation. In this report we examined how often Foxp3^+^ and Foxp3^-^ thymocytes express TCRs that can also be expressed by colonic Tregs with low and high levels of Nrp-1 or Helios. Most notably we found that whereas TCRs on CD4^+^Foxp3^-^ and CD4^+^Foxp3^+^ thymocytes overlapped only marginally, many TCRs expressed on colonic Foxp3^+^ T cells with low or high expression of Nrp-1 or Helios were shared. Our findings that Tregs expressing similar TCRs can have different levels of Nrp-1 and different origin agreed with other report, which also found that tTreg and iTreg subsets with low or high expression of Nrp-1 respectively, displayed comparable TCR repertoires and each could completely fill the same Treg-cell niche [58]. In contrast, two other studies found that Nrp-1^low^ colonic Tregs shared only 8 and 9.1% of their CDR3 amino acid sequences with conventional T cells (Tconv) and tTregs, respectively [59]. Similar estimates were also made from analysis of TCRs from peripherally generated pTregs [60]. However, the global diversity of the repertoires studied in the above mentioned reports is disproportionally high as compared to the size of the samples that were compared. Therefore these investigations barely sampled the content of these repertoires, thereby weakening the conclusiveness of these studies.

Indirectly, our results also suggest that the antigenic specificities and the role of pTregs and tTregs in maintaining tolerance overlap and that these subsets do not have specialized suppressive functions adapted to specific immunological milieus or inflammatory settings. In our view tTregs control immune homeostasis and a broad spectrum of autoimmune responses, as well as inflammation whereas pTregs are recruited to complement tTregs and to replenish their repertoire as tTregs differentiation decays with age. Our results do not contradict the reports that Helios and Nrp-1 can influence Tregs function. Reportedly, Helios^high^ Tregs had stronger suppressive capacity and better lineage stability [61, 62], because Helios *per se* may regulate IL-2 production in Tregs by epigenetic silencing of the IL-2 gene [63]. Similarly, Tregs retrieved from Nrp-1-deficient mice were less suppressive than the respective Tregs from wild type mice, and blocking Nrp-1 abrogates suppression of proliferation of responder cells by Tregs [31]. Nrp-1 also enhances the interactions between Tregs and DCs and promotes the activation of the latent form of TGFβ [64]. Finally, expression of Nrp-1 on Tregs was required to limit anti-tumor immune responses and to cure established inflammatory colitis, whereas Nrp-1-induced transcriptome promoted Tregs stability and prevented these cells from further differentiation [29, 65]. Thus, whereas low surface expression of Helios and Nrp-1 does not exclusively label pTregs cells, these molecules can play a specific roles in Tregs activation, tissue-specific homing and marks Tregs subsets with non-overlapping pattern of response or production of specific cytokines [64, 66, 67]. Polyclonal tTregs effectively suppress Th1 and moderately Th2 cells, but Th17 effectors are only minimally suppressed by this subset [68]. CD4^+^Nrp-1 deficient T cells tend to differentiate to overtly autoreactive Th17 effectors and in wild type mice Nrp-1 may direct these cells reprogramming to pTregs, but how often pTregs recruited through this pathway retain or loose Nrp-1 expression is not clear. Likely, the circumstances in which naive or effector CD4^+^ T cells underwent conversion to pTregs determine their phenotypic features, including the expression levels of the two molecules discussed in this report.

## Materials and Methods

### Mice

A^b^EpFoxp3^GFP^ (A^b^Ep), Aire^-^Foxp3^GFP^ (Aire^-^), CNS1^mut^Foxp3^GFP^ (CNS1^mut^) and *Sf*Foxp3^GFP^ (*Sf*) strains were obtained by mating A^b^Ep [42], Aire-deficient (lacking Aire-dependent, tissue-specific antigens expression [69]), Foxp3^CNS1mut^ (received from Dr. Susan Schlenner (KU Leuven, Belgium)) or B6.Cg-Foxp3sf/J (JAX Laboratory) mice, respectively, with C57BL/6Foxp3^GFP^ mice (C57BL/6) [50]. TCR^mini^Foxp3^GFP^ (TCR^mini^) mice were obtained by mating C57BL/6Foxp3^GFP^ with original TCR^mini^ mice [52]. To eliminate expression of the endogenous TCR-α chains, all TCR^mini^ mice were crossed with mice deficient in endogenous TCR-α loci (JAX laboratory) and were heterozygous for the TCR-α Vα2Jα26(Jα2) mini-locus to ensure expression of a single TCR-α chain per T cell. *Sf* (and respective control C57BL/6) mice were used at ages 3–4 weeks old and all remaining animals were 8–12 weeks old. Only males with mutated Sf or CNS1 (both loci are on X chromosome) and males and females from other strains were used. All mice were housed under specific pathogen-free (SPF) conditions, in room temperature; 22°C, 12:12-h light: dark cycle, and had free access to sterilized food and water. Animal welfare was monitored twice daily by assessment of clinical conditions. Mice were euthanized using CO2 gas followed by decapitation.

### Ethics statement

Animals were housed in the Georgia Regents University animal facility in accordance to Institutional regulations. Presented here research has been approved by Georgia Regents University Institutional Animal Care and Use Committee.

### Isolation of thymocytes and T cells from organs

Single-cell suspensions were prepared from the thymus, inguinal and mesenteric lymph nodes by mechanical disruption. Colons were opened longitudinally and contents were flushed with ice-cold HBSS (Cellgro). Subsequently they were cut into small pieces and washed with HBSS supplemented with 5% FCS (HyClone) and 2mM EDTA at 37°C. A single-cell suspension was obtained after treatment with collagenase D (1.0mg/ml) and DNase I (0.1mg/ml) (both from Roche). A purified and concentrated suspension of lymphocytes was obtained after centrifugation on Percoll (GE Healthcare) gradient (45% and 70%). The interface, enriched in leukocytes, was collected and used for experiments.

### Flow cytometry and cell sorting

Thymocytes and T cells were stained with antibodies against CD4, CD8, Helios, Foxp3 or GFP (BD Bioscience, eBioscience or BioLegend), unconjugated Nrp-1 (R&D Systems) and fluorophore-conjugated rabbit anti–goat IgG (Invitrogen), and analyzed using a BD FACS Canto (BD Biosciences). Intranuclear staining for Helios and Foxp3 was performed using the eBioscience kit according to the manufacturer’s instructions. Cells were sorted using MoFlo cell sorter (Beckman Coulter), with 99% purity.

### High-throughput CDR3 sequencing

Analysis of the TCR^mini^ Vα2Jα26(Jα2) CDR3 regions was performed from sorted CD4^+^Foxp3^+^Nrp-1^high/low^ and CD4^+^Foxp3^+^Helios^high/low^ T cells (purity 97%). RNA from Nrp-1 populations was isolated using RNeasy Mini Kit (Qiagen). RNA from Helios populations was isolated using RNeasy FFPE Kit (Qiagen). RNA was converted to cDNA (SuperScript III, Invitrogen) with a Cα-specific primer (5’-TCGGCACATTGATTTGGGAGTC-3’). TCR-α CDR3 regions were amplified using primers with incorporated barcodes (Vα2IT, 5’-CCATCTCATCCCTGCGTGTCTCCGACTCAGTCTCAGCCTGGAGACTCAGC-3’; CαIT, 5’-CCTCTCTATGGGCAGTCGGTGATTGGTACACAGCAGGTTCTGGGT-3’), and the PCR product was sequenced by EdgeBio/BioServ (Gaithersburg, MD). CDR3 regions sequenced on the same chip and derived from different subsets were discriminated based on barcodes, which were validated for optimal performance with the Ion Torrent PGM. Data was analyzed using the custom made CDR3 extraction program (that uses BLAST for sequence comparison to locate known V and J regions in high volumes of sequencing data), and evaluated with statistical methods as previously described [16, 70]. Briefly, MII similarity index was used that measures pairwise similarities between populations by considering the overlap and relative abundances of TCRs.

### Statistical analysis

Statistical significance was determined based on p-value of the two-sided t-test. The index measuring pairwise similarities between populations by considering the overlap and relative abundances of TCRs, as described in [16, 70] was calculated using R package ‘divo’ developed by our group (cran.r-project.org/package = divo).

## Supporting Information

S1 Fig Proportions of CD4^+^Foxp3^+^ thymocytes in C57BL/6, Aire^-^ and “single peptide” A^b^Ep mice. Bars show the mean % of cells expressing high level of Foxp3 (+/- SD) and represent data from three mice.
(TIF)

S2 Fig Gating strategy for analysis of CD4^+^Foxp3^+^ T cells with different expression levels of Nrp-1 or Helios. (A) CD4^+^Foxp3^+^ cells were gated for Nrp-1^high^ and Nrp-1^low^ populations. (B) CD4^+^Foxp3^+^ cells were intracellularly stained for Foxp3 and Helios, and gated for Helios^high^ and Helios^low^ populations. Sample shown is from peripheral lymph nodes.
(TIF)

S3 Fig Proportions of Nrp-1^high^ and Helios^high^ Tregs in “single peptide” mice. Expression of Nrp-1 (A) and Helios (C) on CD4^+^Foxp3^+^ cells from C57BL/6 and “single peptide” A^b^Ep mice in indicated organs. (B, D) Bars show the mean % of cells expressing high level of respective marker (+/- SD), and represent data from three to five mice.
(TIF)

S4 Fig Total number of CD4^+^ and Treg cells in indicated organs from mouse strains analyzed in Figs 1–4. (A-D) Total number of CD4^+^ (left panel) and Treg (right panel) cells. Bars show the mean value of total number of cells (+/- SD), and represent data from three to five mice.
(TIF)

S5 Fig Sequences used for preparation of heat maps in Fig 5. (A-D) 50 most dominant sequences used to prepare heat map in Fig 5 (A-D respectively).
(TIF)
