# Supplementary figures and images for: Differences in Expression Level of Helios and Neuropilin-1 Do Not Distinguish Thymus-Derived from Extrathymically-Induced CD4+Foxp3+ Regulatory T Cells
Source: PLoS One. 2015 Oct 23;10(10):e0141161. doi: 10.1371/journal.pone.0141161 (PMC4619666; doi:10.1371/journal.pone.0141161)

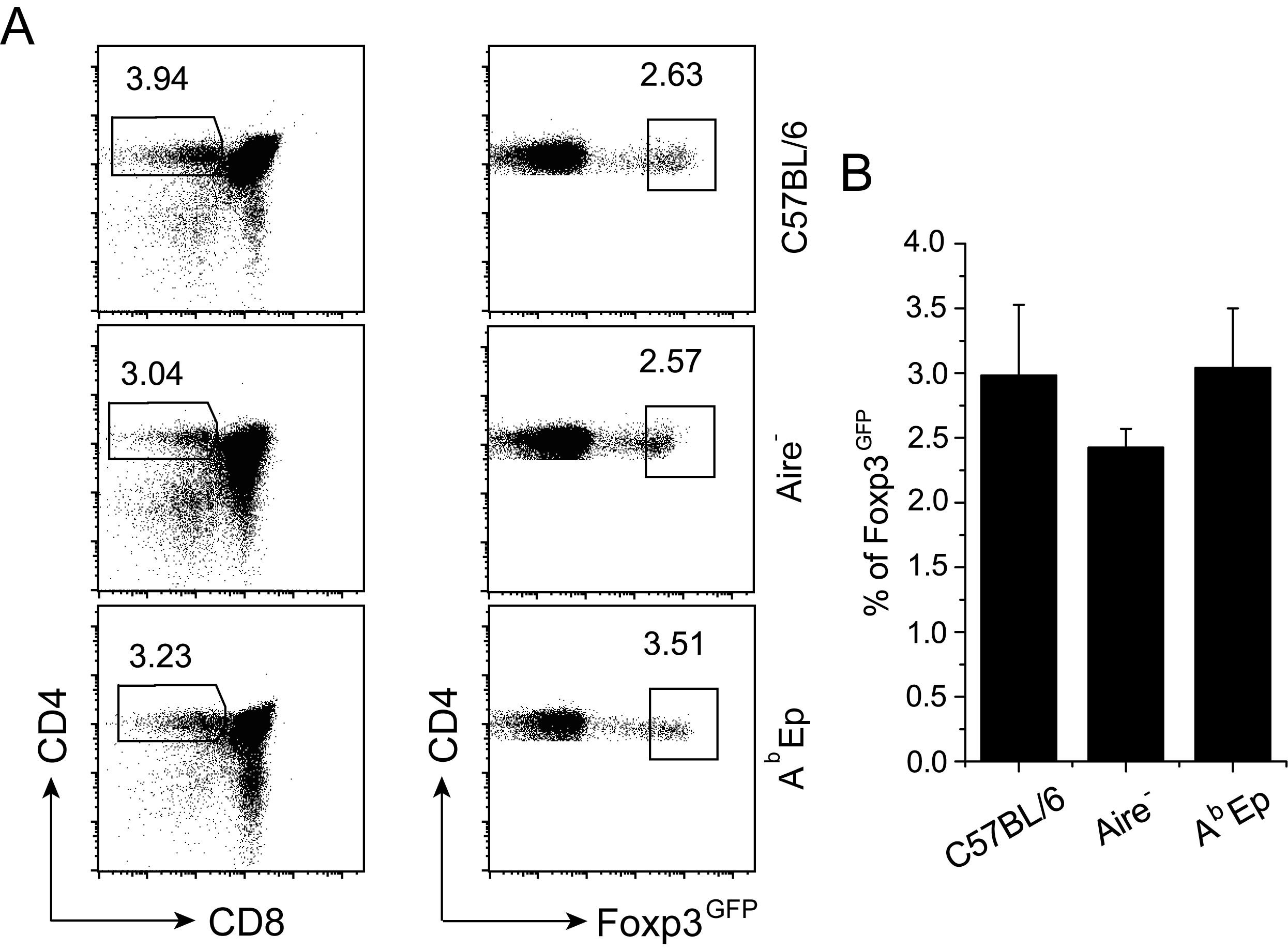

Supplement: S1 Fig — Bars show the mean % of cells expressing high level of Foxp3 (+/- SD) and represent data from three mice. (TIF) [file pone.0141161.s001.tif]

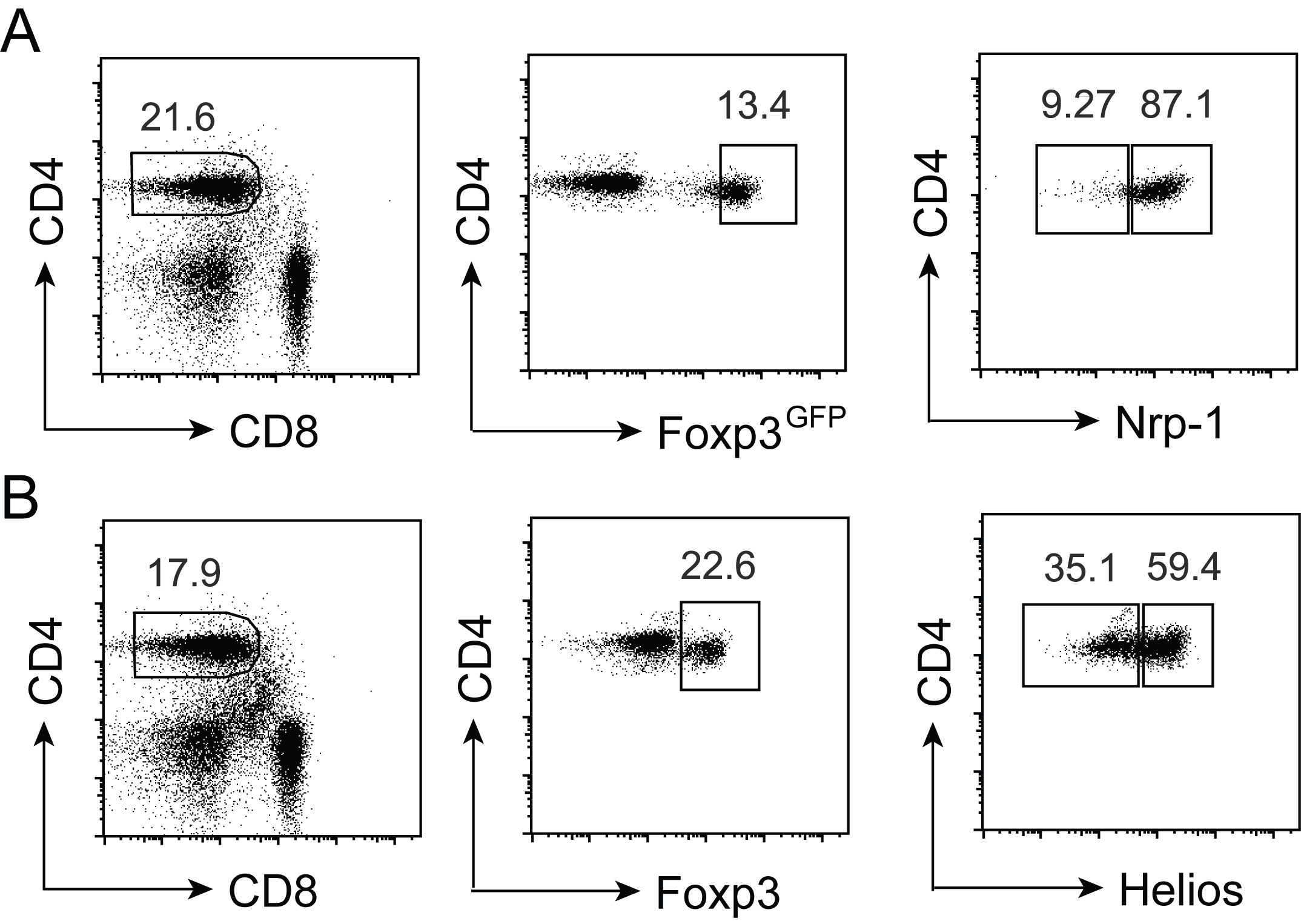

Supplement: S2 Fig — (A) CD4+Foxp3+ cells were gated for Nrp-1high and Nrp-1low populations. (B) CD4+Foxp3+ cells were intracellularly stained for Foxp3 and Helios, and gated for Helioshigh and Helioslow populations. Sample shown is from peripheral lymph nodes. (TIF) [file pone.0141161.s002.tif]

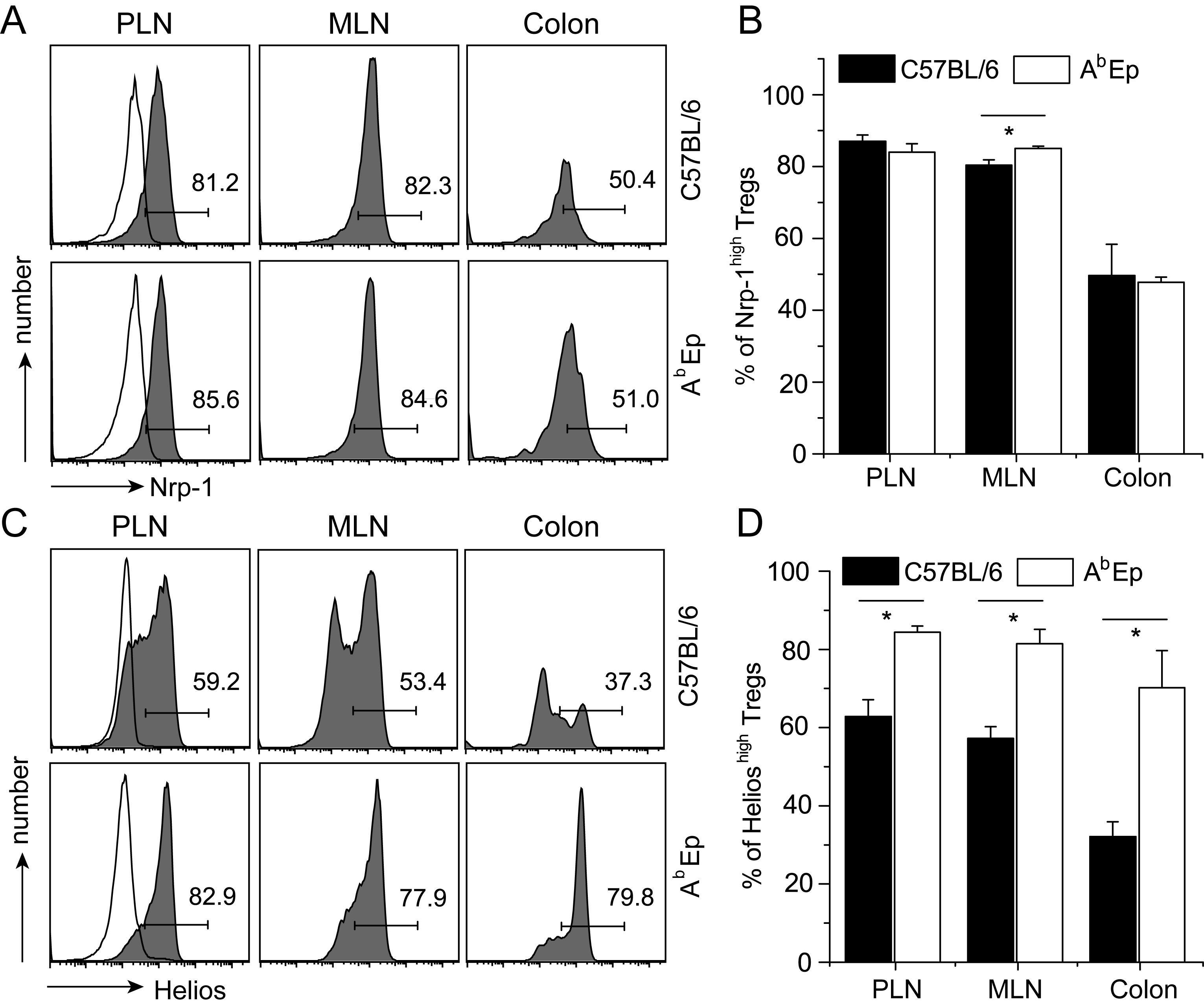

Supplement: S3 Fig — Expression of Nrp-1 (A) and Helios (C) on CD4+Foxp3+ cells from C57BL/6 and “single peptide” AbEp mice in indicated organs. (B, D) Bars show the mean % of cells expressing high level of respective marker (+/- SD), and represent data from three to five mice. (TIF) [file pone.0141161.s003.tif]

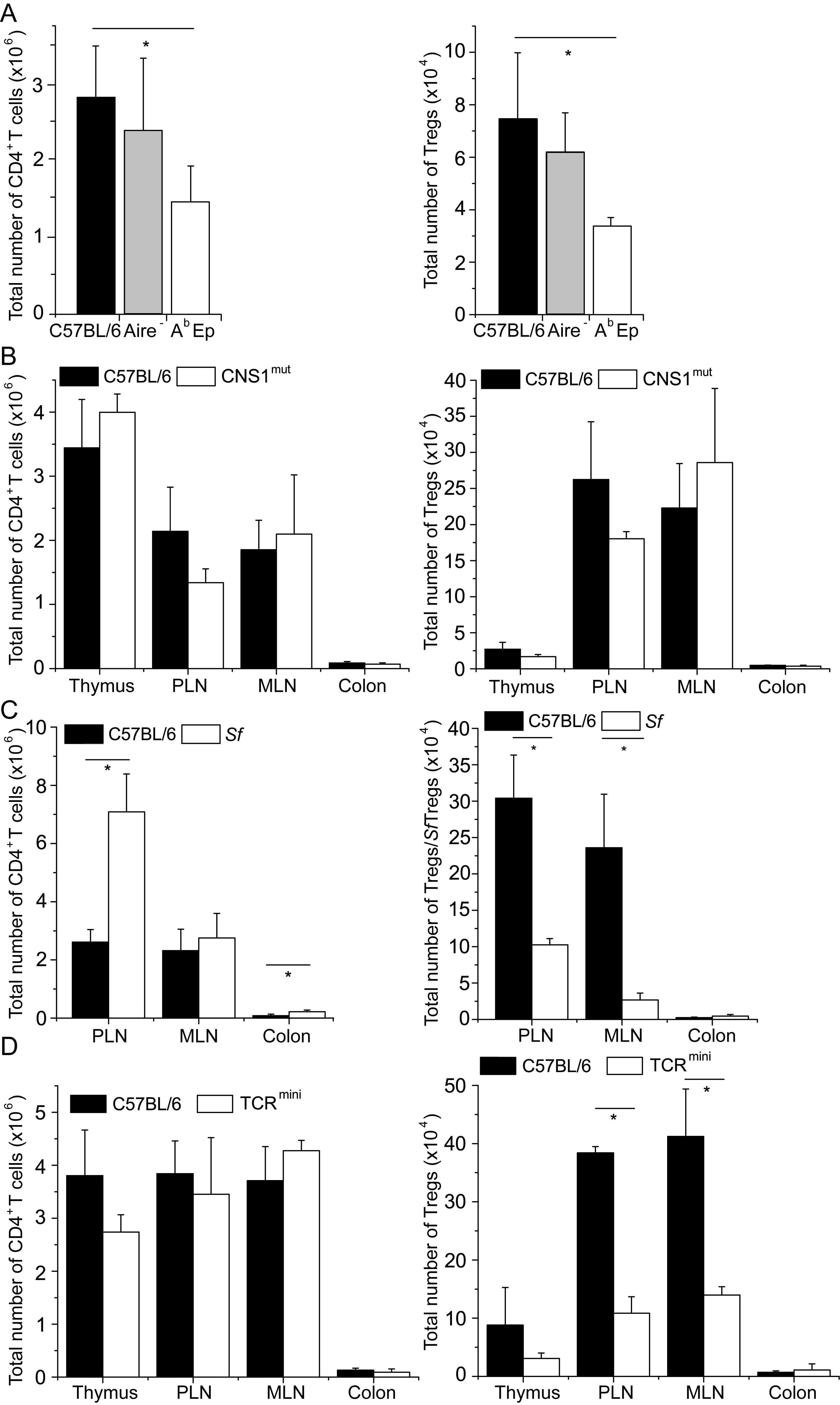

Supplement: S4 Fig — (A-D) Total number of CD4+ (left panel) and Treg (right panel) cells. Bars show the mean value of total number of cells (+/- SD), and represent data from three to five mice. (TIF) [file pone.0141161.s004.tif]

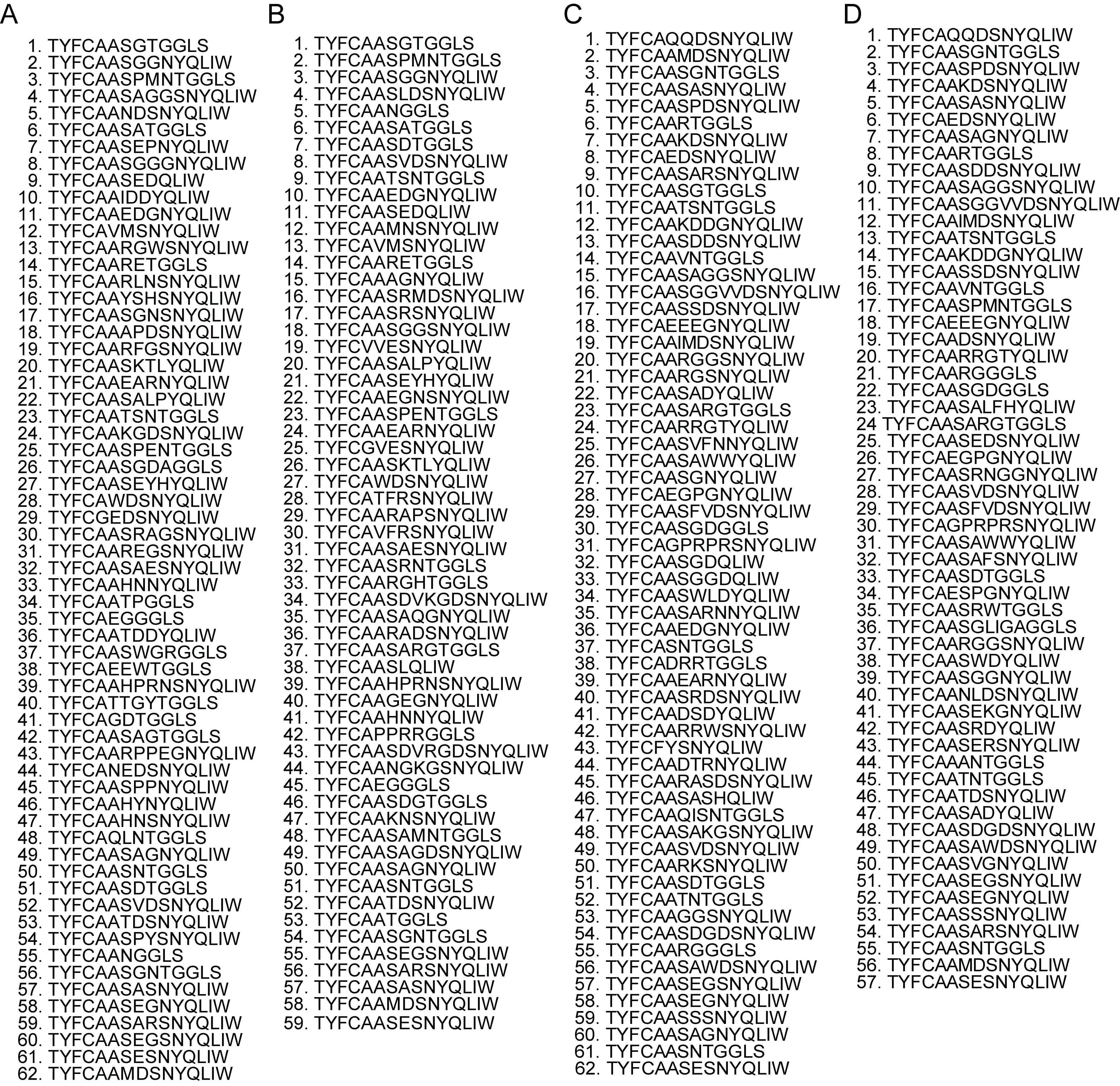

Supplement: S5 Fig — (A-D) 50 most dominant sequences used to prepare heat map in Fig 5 (A-D respectively). (TIF) [file pone.0141161.s005.tif]
